# Surveys of *Drosophila suzukii* (Diptera: Drosophilidae) and Its Host Fruits and Associated Parasitoids in Northeastern China

**DOI:** 10.3390/insects13040390

**Published:** 2022-04-15

**Authors:** Jue Wang, Yanan Zheng, Lichun Fan, Weitao Wang

**Affiliations:** College of Forestry, Shenyang Agricultural University, Shenyang 110866, China; wjgz173@163.com (J.W.); fanlichun17@163.com (L.F.); wangweitao418@163.com (W.W.)

**Keywords:** *Drosophila suzukii*, seasonal occurrence, host fruits, parasitoids

## Abstract

**Simple Summary:**

*Drosophila suzukii* has become a globally invasive pest of thin-skinned berries and stone fruits such as strawberries, blueberries, raspberries, blackberries, and cherries. *D. suzukii* has caused severe economic losses to the fruit industries in more than 30 countries and has been listed as an important quarantine pest in many countries around the world. To better understand the ecology of this invasive pest for its effective management, it is essential to investigate the occurrence of *D. suzukii* and its wild host fruits and natural enemies in its native range. Here, we report the occurrence of *D. suzukii* and its wild host fruits and associated parasitoids in Liaoning, Northeast China for the first time. Four species of wild berries from non-crop habitats were found infested by *D. suzukii*, and two species of parasitoids (*Leptopilina japonica* and *Asobara japonica*) were discovered. Over the survey period from June to October, *D. suzukii* adult populations increased and peaked in August, and then declined until it was no longer detectable in October.

**Abstract:**

Spotted-wing drosophila, *Drosophila suzukii* (Matsumura), is a worldwide quarantine pest that is currently undergoing a rapid range expansion in the Americas, Europe, and parts of Africa. It feeds and breeds on soft-skinned fruits such as raspberries, blueberries, and cherries, and can cause significant economic losses to fruit production. This study investigated the occurrence of *D. suzukii* and its wild host fruits and parasitoids in Liaoning, Northeast China for the first time. Sentinel traps were used to monitor *D. suzukii* adults, and suspected fruits were collected weekly in four different locations (Wafangdian, Faku, Fengcheng, and Shenyang). The results showed that *D. suzukii* were distributed in the sweet soft-skinned fruit-production areas of Liaoning, and raspberry was the most infested fruit. During the field survey, four species of wild berries from non-crop habitats were found infested by *D. suzukii*, and two species of parasitoids (*Leptopilina japonica* and *Asobara japonica*) were collected. *D. suzukii* adult-population dynamics throughout the survey period (June to October) were similar in different survey locations; adult fly populations increased and peaked in August, and then declined until the fly was no longer detectable in October.

## 1. Introduction

*Drosophila suzukii* (Matsumura) (Diptera: Drosophilidae) has become a globally invasive pest of berries and stone fruits, causing significant economic losses to the fruit industry in its invaded regions [1,2,3,4]. The native range of *D. suzukii* is probably East Asia. It was first described by Matsumura in Japan in 1931 [1], and has since been reported in eastern China [5], the Korean Peninsula [6,7], Myanmar [8], Thailand [9], and other regions in southeastern Asia [10,11]. The focus on economic losses due to *D. suzukii* in Japan has been concentrated on cherry and blueberry [1]. However, *D. suzukii* has not been considered a serious fruit pest in other southeastern Asia regions, despite recent frequent reports of damage of soft-skinned fruits by *D. suzukii* in cherry in China [12,13].

In North America, *D. suzukii* has invaded most soft-skinned fruit-growing regions in the US and Canada [2,14,15,16]. The pest caused significant economic losses leading to 100%, 80%, 40%, and 70% losses in cherry, strawberry, blueberry, and raspberry, respectively, in the US in 2008 [16,17]. *D. suzukii* was also detected in Italy, France, and Spain in 2008, and subsequently reported in other European countries [3,18]. Furthermore, in 2013, the fly was on the European and Mediterranean Plant Protection Organization (EPPO) A2 list of pests recommended for regulation [19,20]. *D. suzukii* has since been reported in South American and North African countries [21,22].

Most *Drosophila* species are attracted primarily to damaged, over-ripened, rotted, or fermented fruit; however, the *D. suzukii* female adult has a prominent, serrated ovipositor that enables the laying of eggs in ripening or unripe fruits [23,24]. The larvae hatch and grow inside the fruit, feeding on the pulp and causing secondary infection by saprophytes [16], leading to a loss in the quality and commercial value of the fruits.

*D. suzukii* is a highly polyphagous pest and affects many economically important fruit crops [25,26,27], such as cherry, raspberry, blackberry, blueberry, peach, strawberry, grapes, and other soft-skinned fruits [28,29]. In addition, there are more than 60 wild host plants for *D. suzukii*, such as *Mahonia aquifolium* (Pursh) Nutt. (Berberidaceae), *Frangula purshiana* (DC.) A.Gray ex J.G.Cooper (Rhamnaceae), and *Sambucus williamsii* Hance (Adoxaceae) [26].

Insecticides have been widely used in the control of *D. suzukii* [17,18,30]. However, spraying insecticides may not kill the larvae feeding inside fruits, and *D. suzukii*’s fast development and high reproductive capacity [31,32,33] can result in a rapid population increase [34,35]. More importantly, frequent spraying promotes resistance to pesticides [36], increases insecticide residues on fruits, leads to pest resurgence, affects natural enemies and pollinators [37], and causes secondary pest outbreaks [16,17], affecting control efforts. To safely and effectively control *D. suzukii*, the sustainable integrated pest-management (IPM) approach of combining biological, chemical, and cultural control was proposed to reduce the sole reliance on insecticides. Biological control, as a self-perpetuating control option, is an important part of IPM. The introduction of parasitoids to control *D. suzukii* population densities provides a new tool for reducing pesticide risk and is an environmentally friendly management strategy in crop fields and non-crop habitats.

China is one of the native ranges of *D. suzukii*, and *D. suzukii* was first recorded in 1937 [5]. Parasitoids of *D. suzukii* in Northeast China, a major region planting small berries, has not yet been studied. Previous surveys of parasitoids of *D. suzukii* have been conducted in Southwestern China (Kunming provinces) [38], and only limited surveys have been conducted in a few other provinces (Beijing, Hubei, Sichuan and Jilin) [38]. To introduce and release natural enemies, especially host-specific and effective parasitoids from the native region of its host pest to the pest’s invaded regions, will be an helpful supplement of IPM. Information on the occurrence and host plants of this pest in its native range is still limited. Therefore, we investigated the occurrence of *D. suzukii* and its host plant and associated parasitoids in Northeast China, one of the largest small-berry-planting areas of China. It may provide information on natural enemy recourse for biological control of the pest in Europe, the Americas, and Africa.

## 2. Materials and Methods

### 2.1. Survey Locations

*D. suzukii* were collected from seven sites at five different locations: Wafangdian (WFD), Fengcheng (FC), Shenyang (SY), Faku (FK), and Fushun (FS) in Liaoning province, northeast China (Table 1).

### 2.2. Collections of D. suzukii and Parasitoids

Surveys were carried out during the fruiting season for 17 consecutive weeks from June to October 2016. Twelve sentinel traps were placed (six yeast-sugar-baited traps and six uninfested-fruit-baited traps) in each collection site in the commercial or research farms. Each yeast-sugar-baited trap was placed at a height of 1–1.5 m above the ground, and the uninfested-fruit-baited trap was placed under the yeast-sugar-baited trap and covered with a lid to block direct sunlight or rain. The yeast-sugar-baited trap was made from a 1000 mL transparent plastic container (Horeca select, CN) with 20 holes (0.5 cm diameter) around the side and was filled with 300 mL of bait (21.5 g yeast, 43 g sugar, and 200 mL water). The uninfested-fruit-baited trap was made from a 500 mL plastic container (Horeca select, CN) with 10 holes (0.5 cm diameter) around the side. It was filled with uninfested fruits (banana, blueberry, and raspberry) and agar hydrocolloid (0.8%). The traps were replaced once a week and taken to the laboratory of Forestry College, Shenyang Agricultural University to sort out, count, and record the numbers of larvae, pupae, and adults of flies. Larvae and pupae of *Drosophila* were placed in a 500 mL transparent plastic container (Horeca select, CN) with an artificial diet cultivated at 22 °C and 70% relative humidity (RH). All adults were preserved separately in 75% ethanol for identification.

Both ripe cultivated fruits next to each sentinel trap in the commercial and research fields and ripe wild berries in natural habitats were randomly collected as potential host fruits and taken to the laboratory. Next, the fruits were weighed and placed in 500 mL plastic containers (Horeca select, CN) and incubated at 22 °C and 70% (RH). Fruit juice was constantly removed to prevent the emerged larvae from drowning. The resultant *D. suzukii* pupae were identified from the rotten fruits under a microscope; then, the pupae were individual placed in 1.5 mL Eppendorf tubes at 22 °C and 70% (RH) and checked for emerging parasitoids every day. Furthermore, the flies and parasitoids were preserved in 75% alcohol for identification.

### 2.3. Morphological Identification of D. suzukii and Parasitoids

Specimens of *D. suzukii* were morphologically identified according to the key and descriptions by Okada [9] and Bock & Wheeler [39]. *Drosophila* samples were identified based on morphology of body color, body length, wings, male’s tarsal comb, and ovipositor by dissecting and observing under a microscope. Parasitoid morphological identification was carried out according to the description by Guerrieri et al. and Abram et al. that provided pictures of the genus Leptopilina spp. [40] and Asobara spp. [41]. The figitids were further confirmed by Dr. Matthew Buffington (USDA-ARS, Systematic Entomology Laboratory, Washington, DC, USA). Each parasitoid specimen was examined for its body color, body length, wings, antenna, and leg morphology under a microscope.

### 2.4. Statistical Analysis

All analyses were conducted using the Statistical Package for Social Sciences software (SPSS20.0 version, SSPS Inc, Chicago, IL, USA), and one-way analysis of variance (LSD multiple comparisons, *p* < 0.05) was used to compare different treatments. The relative abundance and sex ratio of *D. suzukii* were calculated as follows:

Relative abundance (RA) = (the amount of *D. suzukii*/the total amount of Drosophilidae) × 100%

Female ratio = (the amount of *D. suzukii* females/the amount of all *D. suzukii*) × 100%

## 3. Results

### 3.1. Morphological Characteristics of D. suzukii and Parasitoids

**Figure 1:**
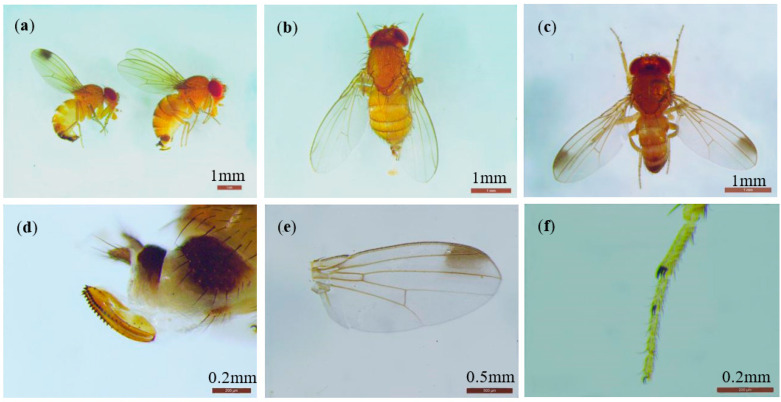

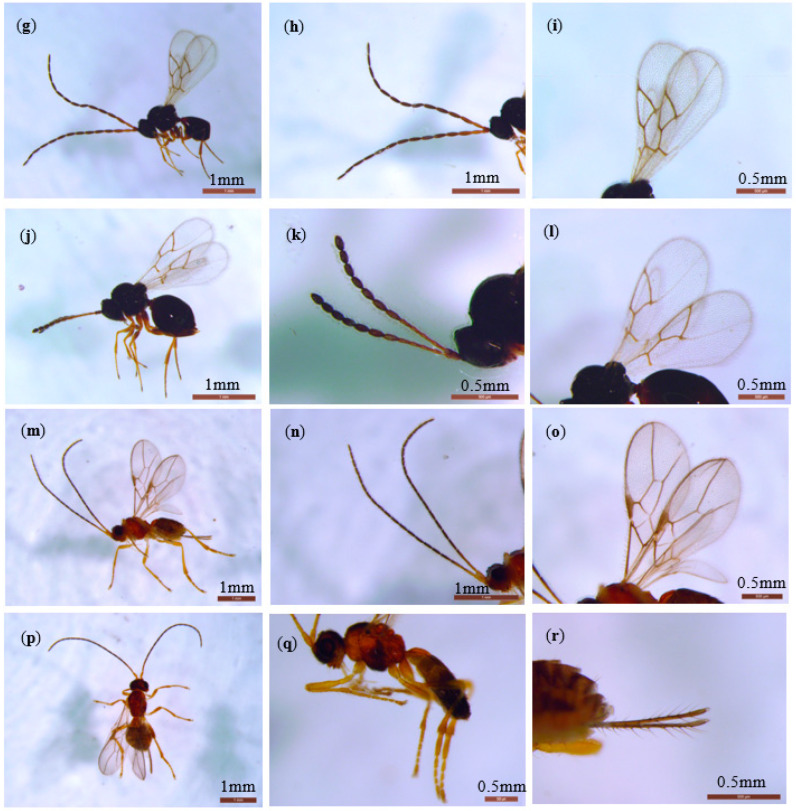
Morphological identification of *D. suzukii* adults, *L. japonica* adults, and *A. japonica* adults. (**a**–**f**): *D. suzukii* adult. (**a**) adult male and female (relative body size), (**b**) adult female, (**c**) adult male, (**d**) female ovipositor, (**e**) male wing with a black spot, (**f**) the sexual combs on male’s fore tarsi. (**g**–**l**): *L. japonica* adult. (**g**) male adult, (**h**) male antennae (15 segments), (**i**) male forewings, (**j**) female adult, (**k**) female antennae (13 segments), (**l**) female forewings. (**m**–**r**): *A. japonica* adult. (**m**) female adult, (**n**) female antennae, (**o**) female forewings, (**p**) the dorsum of female, (**q**) the mesosoma and metasoma of female, (**r**) female ovipositor.

*D. suzukii* were collected at all survey sites (Figure 1). Three parasitoids emerged from *D. suzukii* pupae from raspberries collected in FK. Two of them were identified as Leptopilina japonica Novković & Kimura (Hymenoptera: Figitidae) (1♀1♂) and one was Asobara japonica Belokobylskij (Hymenoptera: Braconidae) (♀) (Figure 1).

### 3.2. Captures of Adult D. suzukii in Sentinel Traps

A total of 406 traps were deployed and successfully recovered from four different locations, and *D. suzukii* adults were found in all locations. In total, 11,229 adults of *D. suzukii* (6697 females and 4532 males) and 34,743 adults of other *Drosophila* species were captured.

**Figure 2:**
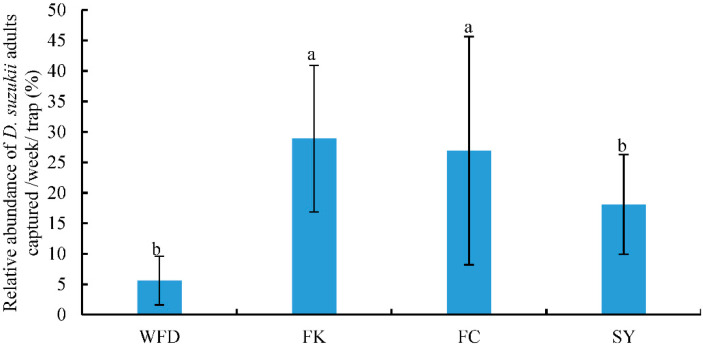
Relative abundance (mean ± SE) of *D. suzukii* adults captured from different locations. Different letters above the bars indicate a significant difference between locations (*p* < 0.05, LSD).

Different adult *Drosophila* species were captured from survey locations, and *D. melanogaster* Meigen was the most abundant species. The relative abundance (RA) of *D. suzukii* adults was less than 30% at all survey locations. The RAs of *D. suzukii* adults captured were 5.61%, 28.89%, 26.91%, and 18.10% from WFD, FK, FC, and SY, respectively (Figure 2, Supplementary Data S1). The RAs of *D. suzukii* adults captured from FK and FC were significantly higher than those from WFD and SY (F = 7.720, df = 3, 37, *p* < 0.05).

**Figure 3:**
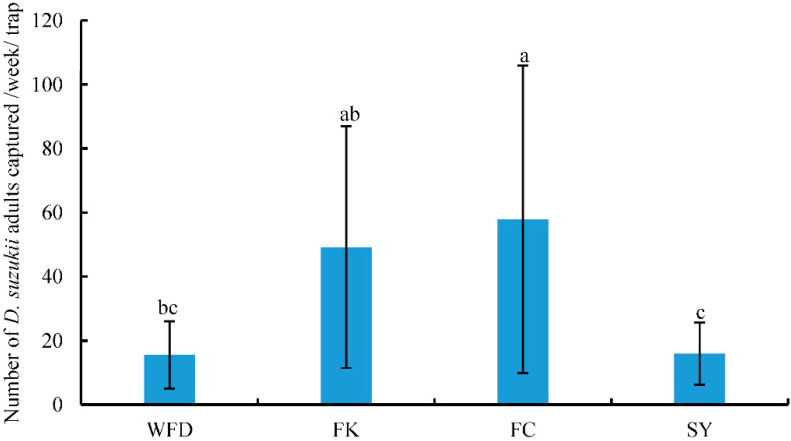
Number (mean ± SE) of *D. suzukii* adults captured per week per trap at different locations. Different letters above the bars indicate a significant difference between locations (*p* < 0.05, LSD).

In FC, a weekly average of 57.86 *D. suzukii* adults was captured, which was the highest compared to other survey locations and was significantly higher than those from WFD or SY. Conversely, WFD recorded the lowest (15.56) average weekly amount of *D. suzukii* adults captured in each trap compared to FK and FC and the difference was significant (Figure 3, Supplementary Data S1) (F = 1.661, df = 3, 37, *p* < 0.05).

**Figure 4:**
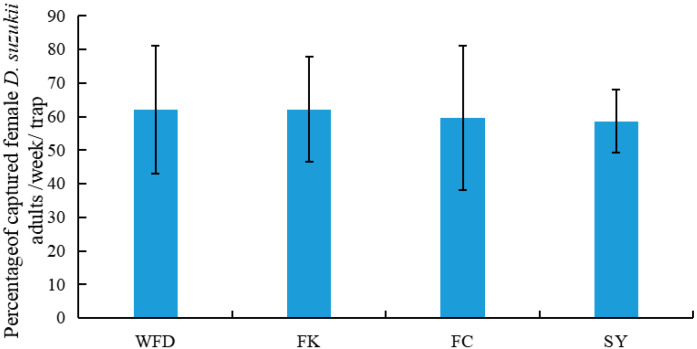
Weekly percentage (mean ± SE) of captured female *D. suzukii* adults at different locations.

Among the survey locations, the sex ratio of *D. suzukii* adults captured was not significantly different and ranged between 55% and 65% (WFD: 62.06%, FK: 62.18%, FC: 59.69%, SY: 58.65%) (Figure 4, Supplementary Data S1) (F = 0.073, df = 3, 37, *p* > 0.05).

**Figure 5:**
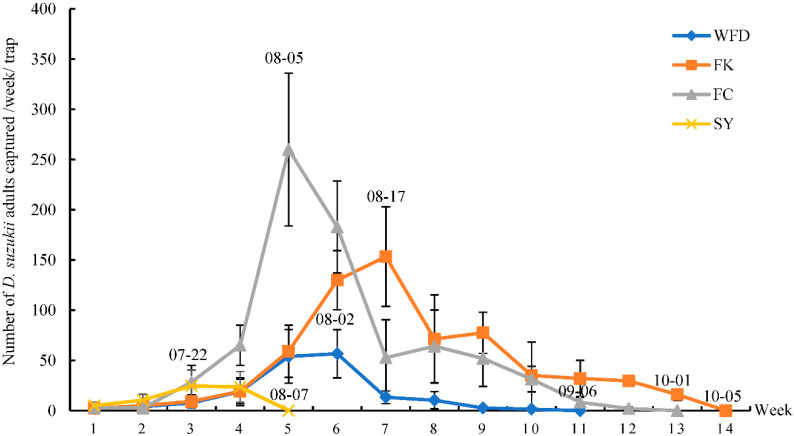
Mean number of *D. suzukii* captured per week per trap at the different locations. The numbers on the curves indicate the sampling date (month-day). In WFD, 1–11 weeks start from 06-21 to 09-06. In FK, 1–14 weeks start from 06-22 to 09-28. In FC, 1–13 weeks start from 06-26 to 10-01. In SY, 1–5 weeks start from 07-02 to 08-06.

The population dynamics of *D. suzukii* adults were similar among the different locations, with one capture peak in August and then a decrease to zero by October. The peak number of *D. suzukii* adults captured per week per trap was 259.88 in FC, significantly higher than those from other locations; and the peak amount of *D. suzukii* adults captured was 24.67 in SY, which was the lowest compared to other locations. The number of *D. suzukii* adults captured reached its peak on 07-22 (month-day), 08-02, 08-05, 08-17, at SY, WFD, FC, and FK, respectively, and decreased to zero on 08-07, 09-06, 10-01, and 10-05, at SY, WFD, FC, and FK, respectively (Figure 5, Supplementary Data S1).

**Figure 6:**
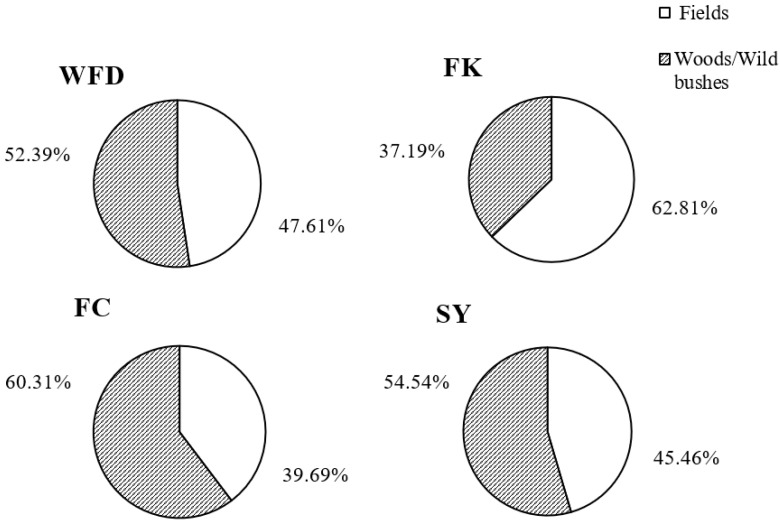
Relative abundance (mean ± SE) of *D. suzukii* adults captured from field and woody habitats.

The relative abundances of *D. suzukii* adults captured were not significant different between commercial fields and woods/wild bushes in WFD (F = 0.001, df = 1, 20, *p* > 0.05), FK (F = 3.985, df = 1, 24, *p* > 0.05), FC (F = 2.178, df = 1, 24, *p* > 0.05), and SY (F = 0.189, df = 1, 6, *p* > 0.05) (Figure 6, Supplementary Data S1).

### 3.3. D. suzukii Larvae Numbers in Suspected Fruits

**Figure 7:**
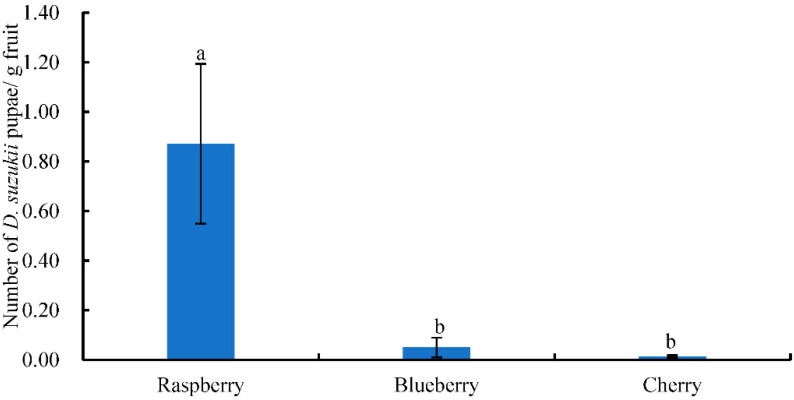
Number (mean ± SE) of *D. suzukii* pupae/g fruit from different fruits. Different letters above the bars indicate a significant difference between fruits (*p* < 0.05, LSD).

*D. suzukii* larvae were found in soft-skinned fruits, such as raspberries, blueberries, and cherries. The total numbers of *D. suzukii* pupae recorded were 4619, 102, and 24 from raspberry, blueberry, and cherry, respectively. The number of *D. suzukii* pupae/g fruit in raspberry was significantly higher than in blueberry or cherry (F = 17.055, df = 2, 48, *p* < 0.05) (Figure 7, Supplementary Data S1).

**Figure 8:**
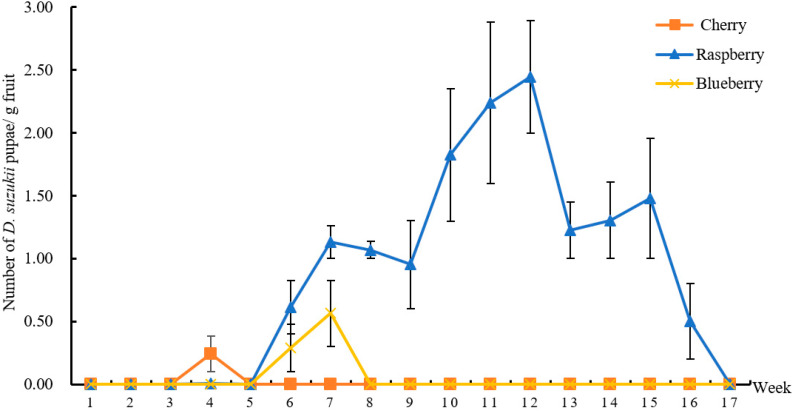
Weekly mean number of *D. suzukii* pupae/g fruit in different fruits. Weeks 1–17 start from 06-28 to 10-31.

In WFD, *D. suzukii* larvae were found in cherries only at the 4th week, which was the peak maturity period for cherries. In FC, *D. suzukii* larvae were found in blueberries at the 6th and 7th week, and many blueberries were damaged due to heavy rains in that period. In FK, *D. suzukii* larvae were found in raspberries during the fruit development period, and *D. suzukii* larvae were present in each collected raspberry. The weekly mean number of *D. suzukii* pupae peaked at the 12th week and then decreased to zero at the 17th week in FK, and the peak number of *D. suzukii* pupae was 2.43/g (Figure 8, Supplementary Data S1).

### 3.4. D. suzukii Surveys in Wild Host Species

Wild berries of 10 different plant species were collected from non-crop habitats in Liaoning, and the list of the plant species from which *D. suzukii* emerged is provided in Table 2.

## 4. Discussion

This was the first study that surveyed the occurrence of *D. suzukii* and its associated host plants and parasitoids in both crop and non-crop habitats in the major berry-fruit-production regions in Northeastern China. Our results showed that: (1) *D. suzukii* occurred in raspberries, blueberries, and cherries, and raspberries appeared to be the most seriously infested crop; (2) four wild berries from non-crop habitats were also infested by *D. suzukii*; (3) two species of parasitoids occurred in Liaoning, China; (4) FC had the highest captures of *D. suzukii* adults in sentinel traps compared to other three survey locations; and (5) throughout the surveyed period (from June to October 2016), the seasonal population dynamics of *D. suzukii* adults were similar in different survey locations.

Many studies have showed that the number of captured *D. suzukii* adults varied among the different geographic locations, the host plant life cycle, temperature, and rainfall [34,42,43]. Like other colder or northern regions in Europe or North America [44], there was only one peak of adult flies per year in Liaoning. Fly populations appeared in June, increased steadily over the summer to reach a peak in August, and flies were no longer detected by October as temperatures decreased. Among the four locations, the numbers of *D. suzukii* adults captured in FK and FC were significantly higher, and collected fruits in these two locations were also more severely damaged than those from WFD and SY. The FK and FC cites were surrounded by woods and bushes, and these non-crop habitats had likely provided source populations of *D. suzukii* and affected the occurrence and distribution of the flies. In FK, the number of *D. suzukii* larvae collected at the end of the harvest period was higher than that of other periods, probably because those unpicked ripe fruits provided breeding sites for *D. suzukii* adults. Furthermore, In FC, the number of *D. suzukii* larvae collected in blueberries during the 4th and 5th weeks was significantly higher than in other weeks. This was likely because blueberries were mechanically injured due to the heavy rains and strong winds, and juices from the overripe, damaged, or split fruits could attract *D. suzukii* adults.

*D. suzukii* has a wide range of hosts, and many fruits are oviposition hosts, adult food sources, or provide shelter for overwintering *D. suzukii* [26]. In addition to commonly cultivated soft-skinned fruits, *D. suzukii* can also infest a variety of wild or ornamental hosts [26]. *D. suzukii* most likely overwinters in forests with wild hosts where refuge and nutrients are more abundant than crop fields. During the spring and early summer, small overwintering populations likely build in non-crop areas to escape monitoring and insecticides [45,46]. Surveying and identifying wild hosts, followed by removal of whole plants or fruits, can reduce *D. suzukii* habitats and help manage *D. suzukii* populations [44]. The wild berries from ten sampled plant species were collected from non-crop habitats in Liaoning, four of which were infested by *D. suzukii*. However, infestations in the field will depend on the level of *D. suzukii* populations, host plants (including fruits ripeness, age, and architecture) and relative attractiveness of other hosts in surrounding vicinity [26]. Therefore, the absence of infestation in the other six fruiting species does not necessarily indicate that they are unsuitable hosts. They are still potential wild hosts for *D. suzukii*, and more extensive investigations are needed in the future. Furthermore, studies on host preference by *D. suzukii* showed that the fly prefers soft-skinned fruits. Therefore, there is a need to determine the kinds of volatile compounds involved, which will be helpful to develop a better trap than the current yeast traps for the monitoring and control of *D. suzukii* in the future.

Many natural enemies, including parasitoids, predators, and entomopathogens, have been evaluated under laboratory conditions for their efficiency against *D. suzukii*, and some of them have the potential to be used for biological control of this pest [47,48]. In particular, some host-specific parasitoids could be promising natural enemies. There are more than 50 hymenopteran parasitoid species worldwide, attacking *Drosophila* species in the larval or pupal developmental stages [49]. Genetic analyses suggest East Asia is the region of origin for the *D. suzukii* populations that invaded North America [50]. As the origin region of *D. suzukii,* East Asia should be the focal region for parasitoid collections [51]. To date, no locally occurring larval *Drosophila* parasitoids can readily develop from *D. suzukii* in the invaded regions. In contrast, 19 species of larval *D. suzukii* parasitoids were collected from *D. suzukii* in East Asia [48]. Among them, *L. japonica*, *Ganaspis brasiliensis* (Ihering), and *A. japonica* were the dominant parasitoids [52,53].

*G. brasiliensis* and *L. japonica* are the most abundant and frequently collected larval parasitoids, and predominantly or exclusively reared from *D. suzukii* with reported highest parasitism rates >70% [54]. At least one genetic group of *G. brasiliensis* was found to be the most host-specific to *D. suzukii*, which has only been collected from fresh fruits infested by *D. suzukii* and other closely related drosophilids [38,54]. Thus, the more host-specific *G. brasiliensis* was currently being considered for introduction into North America and Europe [38]. *L. japonica* was first collected from fresh cherries in Trento, Italy in 2019, and in the coming year, *L. japonica* was collected from more locations there, which confirmed that *L. japonica* is widely established in the region. They shared more than 99% sequence similarity with specimens of *L. japonica* collected in Asia. This means that *L. japonica* was probably accidentally introduced into Italy from Asia [55]. Both *G. brasiliensis* and *L. japonica* were found established in British Columbia in 2020, probably resulting from accidental introduction [56]. Although China is one of the native ranges of *L. japonica*, there are few studies on it. Previous studies have only confirmed the distribution of *L. japonica* in Yunnan, Sichuan, and Beijing in China [38]. Further research is needed on the distribution and biological characteristics of *L. japonica* in China. As one of the native ranges of *D. suzukii*, the Chinese fruit industry has not suffered serious economic losses [2], probably due to the wide occurrence of some effective native natural enemies of *D. suzukii*. At least 10 larval parasitoids, *A. japonica*, *Asobara leveri* (Nixon) (Hymenoptera: Braconidae), *Asobara mesocauda* van Achterberg and Guerrieri (Hymenoptera: Braconidae), *Asobara triangulata* van Achterberg & Guerrieri (Hymenoptera: Braconidae), *Asobara pleuralis* (Ashmead) (Hymenoptera: Braconidae), *Areotetes striatiferus* Li, van Achterberg and Tan (Hymenoptera: Braconidae), *G. brasiliensis*, *L. japonica*, *Tanycarpa chors* Belokobylskij (Hymenoptera: Braconidae), and *Leptopilina* sp. (Hymenoptera, Figitidae), and the two pupal parasitoids, *Pachycrepoideus vindemiae* (Rondani) (Hymenoptera: Pteromalidae) and *Trichopria drosophilae* Perkins (Hymenoptera: Diapriidae) were collected in Sichuan, Yunnan, Beijing, and Hubei Provinces of China [38,53]. The current survey in Liaoning further complemented previous research on the diversity and distribution of these parasitoids in China. However, we only collected *L. japonica* and *A. japonica*, and did not find *G. brasiliensis*. The parasitoid species found in Liaoning were much less diverse than other warmer regions in China as well as in South Korea and Japan [38,52,53]. In the future, more extensive surveys of native parasitoids in East Asia may be needed to discover different species/strains that can establish in different climatic zones in the fly’s invaded regions.

## Figures and Tables

**Table 1:** Sampling locations and sites for *D. suzukii* and its parasitoids in 2016 Liaoning, China.

Survey Location	Collection Site	Host Plant Habitat	Coordinates	Collection Date	Mean Temperature (°C)	Mean Humidity (%)	Mean Rainfall (mm)
Wafangdian	Delisi Orchard	Commercial cherry farm	N 39°47′ E 122°03′	Jun 21–Oct 31	20.51	75.14	2.91
Fengcheng	Enhue Orchard	Commercial blueberry farm	N 40°24′ E 123°57′	Jun 22–Oct 31	19.06	77.54	4.64
Fengcheng	Fenghuangshan	Natural forest	N 40°24′ E 124°4′	Aug 2–Sep 24	21.53	81.15	3.96
Shenyang	Shenyang Agricultural University	Research raspberry farm	N 41°49′ E 123°34′	Jul 2–Aug 26	24.97	63.74	6.93
Faku	Maanshan Orchard	Commercial raspberry farm	N 42°26′ E 122°52′	Jun 26–Oct 31	18.67	74.59	4.21
Fushun	Huangqi City	Natural forest (wild raspberry)	N 41°51′ E 123°54′	Jul 18	22.54	76.16	0.08
Fushun	Wendao Forest	State owned forest	N 41°8′ E 124°2′	Sep 7	19.85	88.11	5.68

Climate data (temperature, humidity, and rainfall) was collected from meteorological stations in China (China Meteorological Administration, 2016. http://data.cma.cn/ (accessed on 24 March 2022)).

**Table 2:** Wild berry species from which *D. suzukii* adults emerged in different non-crop habitats in Liaoning in 2016.

Collection Location ^1^	Collection Date	Wild Berry Species	Was *D. suzukii* Present?
FC	July 8–October 1	*Actinidia arguta* (Sieb. & Zucc) Planch. Ex Miq.	Yes
		*Rubus crataegifolius* Bunge	Yes
		*Lonicera maackii* (Rupr.) Maxim.	No
SY	July 9–August 26	*Bothrocaryum controversum* (Hemsl.) Pojark	No
		*Cerasus tomentosa* (Thunb.) Wall.	No
		*Padus racemosa* (L.) Gilib.	No
		*Rhamnus davurica* Pall.	No
		*Sambucus williamsii* Hance	No
		*Viburnum dilatatum* Thunberg	No
FS	July 18	*Rubus idaeus* L.	Yes
	September 7	*Bothrocaryum controversum* (Hemsl.) Pojark	No
		*Cerasus tomentosa* (Thunb.) Wall.	No
		*Hippophae rhamnoides* L.	No
		*Padus racemosa* (L.) Gilib.	No
		*Rhamnus davurica* Pall.	No
		*Rubus crataegifolius* Bunge	Yes
		*Viburnum dilatatum* Thunberg	Yes

^1^ Collection locations in Liaoning, Northeast China: Fengcheng (FC), Shenyang (SY), and Fushun (FS).

## Data Availability

Data is contained within the supplementary material. The data presented in this study are available in [Supplementary Data S1].

## Supplementary Materials

The following supporting information can be downloaded at: https://www.mdpi.com/article/10.3390/insects13040390/s1, Supplementary Data S1.
